# Size at birth and accelerometer‐measured physical activity or sedentary behavior in healthy term‐born adults

**DOI:** 10.1002/ajhb.23717

**Published:** 2022-01-02

**Authors:** Jessica Leigh Garay, Tiago V. Barreira, Qiu Wang, Tom D. Brutsaert

**Affiliations:** ^1^ Department of Nutrition and Food Studies Syracuse University Syracuse New York USA; ^2^ Department of Exercise Science Syracuse University Syracuse New York USA; ^3^ Department of Higher Education Syracuse University Syracuse New York USA

## Abstract

The present study investigated the relationship between birth size and activity patterns. One hundred and twenty‐four adults wore accelerometers for 7 days. Birth weight was adjusted for gestational age (AdjBW). The overall association between time spent in MVPA/day and AdjBW was not significant (*B* = 5.64, *p* = .09). MVPA/day increased by 7.02 min (*p* = .02) in participants 18–21 years (*N* = 42) and decreased by 10.8 min (*p* = .02) in participants 22–40 years (*N* = 33) per unit increase in AdjBW. The effect of birth size on adult physical activity depends on age.

## 1 INTRODUCTION

Exposure to stressors in utero can lead to diminished fetal growth and development. Low birth weight (LBW), commonly defined as less than 2500 g, is associated with higher fat mass, lower muscle mass, and increased risk of chronic disease throughout life, a phenomenon known as fetal programming (FP; Corvalan et al., 2007; Gluckman et al., 2008). Whether FP impacts behaviors such as patterns of physical activity (PA) or inactivity remains unclear. Humans born small may engage in less PA but results are mixed (Pinto Pereira et al., 2014; Salonen et al., 2011). Historically, PA or sedentary behavior (SED) was determined via questionnaires. Self‐report instruments are subject to recall bias, overestimation of PA, and underestimation of SED (Prince et al., 2008). Past studies using accelerometers have concluded there is no association between birth size and PA, but most were conducted in children and adolescents (Ridgway, Brage, Sharp, et al., 2011). Past studies are split regarding whether they did (Andersen et al., 2009; Azevedo et al., 2008; Ridgway, Brage, Sharp, et al., 2011) or did not (Laaksonen et al., 2003; Pinto Pereira et al., 2014; Ridgway, Brage, Anderssen, et al., 2011; Salonen et al., 2011) account for GA.

The purpose of the current study was to determine if size at birth was associated with moderate‐to‐vigorous PA (MVPA) or SED among healthy young adults. We hypothesized that birth size would be positively associated with MVPA/day and inversely associated with SED/day.

## 2 MATERIALS AND METHODS

### 2.1 Study population

Participants were 124 adults (ages 18–40) selected from an online survey of birth size (*N* = 1246). Inclusion criteria: singleton born at 37–42 weeks gestation; free of current or previous diagnosis of heart disease, diabetes, cancer; current weight maintained for a minimum of 3 months. Exclusion criteria: maternal hypertension or diabetes; underweight (BMI < 18.5) or morbidly obese (BMI >35); current pregnancy, or less than 1‐year post‐partum. The Syracuse University Institutional Review Board approved this study, and participants provided written informed consent.

### 2.2 Measures

A reference dataset was used to predict BW for each participant based on sex and GA (Kramer et al., 2001). The difference between self‐reported and predicted BW (adjusted BW, AdjBW) was calculated and standardized. The AdjBW thus represents each participant's relative BW above or below the overall sample mean BW, taking into account sex and GA.

Participants wore an activPAL (AP) (PAL Technologies Ltd, Glasgow, UK) on the left thigh and an ActiGraph GT3X+, (AG) (ActiGraph LLC, Fort Walton Beach, FL) on the left hip for a continuous 7‐day period, including while asleep, only removing the devices when coming into direct contact with water (i.e., bathing or swimming). AP is a uni‐axial monitor containing an inclinometer to determine limb position. Sampling rate was 20 Hz, and data were downloaded in 15‐s epochs (activPAL3™ software version 7.2.32). The AG device is a triaxial accelerometer, generating activity counts for each axis and a vector magnitude representing the combination of all three axes. In the current study, the data were collected at a frequency of 80 Hz. Details regarding the AG device have been published elsewhere (Barreira et al., 2018).

MVPA was identified from the AG device and defined as greater than 2020 counts/minutes, approximately three metabolic equivalents (METs) or higher (Atienza et al., 2011; Piercy et al., 2018). This value is in line with the 2018 Physical Activity Guidelines for Americans. Time spent in SED was identified from the AP device using the proprietary classification of “sitting/lying.” Consistent with prior research using accelerometers within NHANES, a complete day was defined as at least 10 h of wear time while awake (Atienza et al., 2011). Periods of non‐wear were identified using NHANES definitions (Atienza et al., 2011; Healy et al., 2011). A minimum of 4 days (including at least 1 weekend day) of data was required, consistent with past research (Ward et al., 2005). Days containing a discrepancy of 7000 total steps/day or more between the two devices were discarded.

Weight (kilograms, kg) and body fat were measured using the Tanita SC‐240 digital scale (Tanita Corporation, Arlington Heights, IL). Maximal aerobic fitness level (VO_2max_) was measured on a treadmill using open‐circuit spirometry (Parvo‐medics TrueOne 2400, Sandy, UT). Participants began walking at 3.5 miles per hour (mph), 0% incline. After 2 min (min), speed increased to 5.5 mph. Speed increased by 1.0 mph every 2 min until maximum speed (7.5 mph for women; 8.5 mph for men). Incline then increased by 2.5% every 2 min. The test ended when the participant reached volitional fatigue. At least two of the following criteria had to be met for a valid VO_2max_ test: respiratory exchange ratio ≥ 1.1, heart rate ≥ 85% of age‐predicted maximum heart rate, plateau in VO_2_ (defined as change of no more than 150 mL/min VO_2_ with increasing work).

### 2.3 Statistical analysis

IBM SPSS Statistics version 23 for Windows (SPSS Inc., Chicago, IL) was used for data analysis. All variables were evaluated for normal distribution using standard procedures.

Linear regression analysis was used to determine the association between AdjBW and either min of MVPA or SED per day. Several covariates were considered for inclusion in the regression models, including: sex, age, lean body mass, body fat percentage, aerobic fitness level, (accelerometer) wake wear time (the amount of time the accelerometer was worn while the participant was awake), and maternal smoking. The model for MVPA included sex, age, body fat percentage, VO_2max_, accelerometer wear time, and the interaction term for AdjBW with age. The model for SED included sex, age, body fat percentage, VO_2max_, accelerometer wear time, and the interaction term for AdjBW with sex. Interactions between AdjBW and all covariates were tested. Due to interaction between AdjBW and age, a separate analysis was performed by splitting the participants into two groups, as equally as possible: participants 18–21 years old (*N* = 42) and participants 22–40 years old (*N* = 33). Significance was set at *p* ≤ .05 for main effects.

## 3 RESULTS

Complete data were collected from 75 of the 124 participants initially enrolled. Twenty‐two participants were excluded from data analysis because they either did not complete both study visits (*N* = 5), did not have adequate accelerometer data (*N* = 12) and/or were subsequently found to be ineligible based on the inclusion and exclusion criteria (*N* = 5). An additional 27 participants were excluded from data analysis because they did not meet the criteria for a valid VO_2max_ test. Descriptive characteristics of the 75 participants included in the data analysis (60 females, 15 males) are provided in Table 1. Ten out of 75 participants (6 females, 4 males) were identified as small for gestational age (defined as less than the 10th percentile for birth weight‐by‐gestational age, based on the reference dataset). Participants wore both accelerometers for an average of 6.2 ± 0.80 days (range 4–7 days). Within each day, participants wore the devices for an average of 929 ± 88 min (range 685–1180 min). Table 1 also displays statistics for the accelerometer measures. There were no significant differences between females and males.

**TABLE 1:** Profile of study participants

	Full sample (*N* = 75)	Females (*N* = 60)	Males (N = 15)
Age (years)	23 ± 5 (18–40)	23 ± 5 (18–40)	24 ± 5 (18–36)
Ethnicity (% Caucasian)	76	77	73
Current BMI	23.5 ± 3.1 (18.7–33.5)	23.6 ± 3.4 (18.7–33.5)	23.5 ± 1.83 (20.0–26.0)
Current % Body Fat	25.8 ± 8.4 (11.0–47.5)	28.2 ± 7.7 (14.3–47.5)	16.3 ± 2.8 (11.0–20.2)
Current LBM (kg)	48.4 ± 7.6 (39.4–68.2)	45.1 ± 2.9 (39.4–53.0)	61.8 ± 5.1 (53.2–68.2)
Birth weight (BW) (g)	3484 ± 505 (2353–4763)	3487 ± 483 (2353–4763)	3474 ± 603 (2580–4621)
Gestational age (weeks)	40.0 ± 1.1 (37.9–42.1)	40.0 ± 1.1 (37.8–42.1)	39.9 ± 1.0 (38.0–42.0)
Difference in BW (g)			
Actual BW − Predicted^a^ BW (g)	7.7 ± 479.1 (−948.7 to 1147.7)	37.2 ± 461.3 (−948.7 to 1147.7)	−110.0 ± 545.7 (−886.8 to 837.1)
AdjBW^b^	0.11 ± 0.98 (−1.84 to 2.44)	0.17 ± 0.94 (−1.84 to 2.44)	−0.13 ± 1.11 (−1.72 to 1.80)
Time spent in MVPA/day, ActiGraph (min)	56 ± 24 (15−117)	54 ± 23 (15–117)	65 ± 27 (16–94)
Steps/day, ActiGraph	9675 ± 3357 (4222–18 606)	9530 ± 3428 (4222–18 606)	10 253 ± 3096 (4827–14 610)
Time spent in SED/day, activPAL (min)	614 ± 96 (299–781)	607 ± 96 (299–781)	641 ± 92 (483–773)

*Note*: Values are mean ± standard deviation (range), or percent frequency.

Abbreviations: BMI, body mass index; LBM, lean body mass.

^a^ Based on sex and gestational age, developed from a reference dataset (Kramer et al., 2001).

^b^ AdjBW (adjusted BW) is the *z*‐score of Difference in BW.

Results of the regression analysis are shown in Table 2. The association between min of MVPA/day and AdjBW was not strong (*p* = .09). VO_2max_ was the largest predictor of time spent in MVPA/day, followed by age. Among the “younger” age group (18–21 years old, *N* = 42), time spent in MVPA/day significantly increased by 7.0 min for each unit increase in AdjBW (*p* = .02), which represented 15.3% of the variation in MVPA. Among the “older” group (22–40 years old, *N* = 33), time spent in MVPA/day significantly decreased by 10.8 min for each unit increase in AdjBW (*p* = .02), explaining 18.7% of the variation in MVPA. Full results of this separate regression analysis are available in Tables S1 and S2, respectively.

**TABLE 2:** Association of AdjBW and covariates on time spent in MVPA/day and SED/day

	Time spent in MVPA/day			Time spent in SED/day		
	B (95% CI)	*p*‐value	Partial η^2^	B (95% CI)	*p*‐value	Partial η^2^
AdjBW	5.64 (−0.87, 12.14)	.09	.04	−14.57 (−36.96, 7.82)	.42	.01
Sex	3.83 (−10.61, 18.26)	.60	.00	56.43 (−1.63, 114.49)	.06	.05
Age	−1.14 (−2.02, −0.26)	**.01**	.09	−2.34 (−5.87, 1.19)	.19	.03
Body fat percentage	0.62 (−0.13, 1.36)	.10	.04	0.64 (−2.34, 3.61)	.67	.00
VO_2Max_ (ml/kg/min)	1.58 (0.98, 2.12)	**≤.001**	.30	−2.96 (−5.24, −0.69)	**.01**	.09
Wear Time	−0.01 (−0.06, 0.05)	.81	.00	0.55 (0.33, 0.77)	**≤.001**	.27
AdjBW*age	−1.01 (−1.83, −0.19)	**.02**	.08			
AdjBW*sex				46.88 (4.05, 89.71)	**.03**	.07

*Note*: *N* = 75. MVPA: Overall *R* ^2^ = .41 (Adjusted *R* ^2^ = .35); SED: Overall *R* ^2^ = .39 (Adjusted *R* ^2^ = .32). *P*‐values in bold are significant.

No significant association was detected between min of SED/day and AdjBW. Accelerometer wake wear time and VO_2max_ were both significant predictors of min of SED/day. Performing the regression analysis by sex did not reveal any significant associations between AdjBW and min of SED/day (Table S3).

## 4 DISCUSSION

In the current study, a significant positive relationship between AdjBW and min of MVPA/day was observed among 18–21‐year‐old participants while a significant inverse relationship was observed among 22–40 year old participants. No relationship was detected between AdjBW and min of SED/day. Previous accelerometer‐based studies found no relationship between MVPA or SED when using BW (controlling for GA; Ridgway, Brage, Sharp, et al., 2011). Similar results were found with BW alone (Ridgway, Brage, Anderssen, et al., 2011). These past studies were all conducted in children; the current study is believed to be the first to objectively measure MVPA and SED (using two different devices) among young adults. More studies are needed to measure PA using accelerometers in adult birth cohorts.

Participants in the current study achieved an average of 56 min of MVPA/day and 611 min of SED/day, compared with 6.7 min of MVPA/day and 506 min of SED/day among NHANES participants, respectively (Atienza et al., 2011; Healy et al., 2011).

This study had a small sample size compared to previous birth cohorts (Ridgway, Brage, Anderssen, et al., 2011). The ability to detect a U‐shaped relationship between birth size and PA improves with larger samples (Andersen et al., 2009). We visually inspected our data and found no evidence of a U‐shaped relationship. Additionally, birth information was self‐reported. A strength of the current study was the use of 2 accelerometer devices to objectively measure MVPA and SED.

In conclusion, among healthy adults, the effect of AdjBW on daily MVPA varied by age, while there was no effect of AdjBW on daily SED time. This study adds to the growing literature base suggesting that although FP affects an individual's physiology, the effect it has on adult behaviors may be influenced by age and/or sex. Individuals should be encouraged to engage in adequate MVPA, and to limit SED time, regardless of birth size. Future studies should confirm the role of intrauterine and early life factors on adult physiological markers and objectively measured PA or SED.

## CONFLICT OF INTEREST

The authors have no conflicts of interest to disclose.

## AUTHOR CONTRIBUTIONS

**Jessica Leigh Garay:** Conceptualization (lead); data curation (lead); formal analysis (lead); funding acquisition (equal); investigation (lead); methodology (lead); project administration (lead); writing – original draft (lead); writing – review and editing (lead). **Tiago V. Barreira:** Data curation (equal); formal analysis (supporting); writing – review and editing (supporting). **Qiu Wang:** Formal analysis (supporting); writing – review and editing (supporting). **Tom D. Brutsaert:** Conceptualization (equal); funding acquisition (lead); methodology (supporting); writing – review and editing (supporting).

## Supporting information


**Table S1** Association of AdjBW and select covariates on time spent in MVPA/day among “young” participants aged 18–21.
**Table S2**. Association of AdjBW and select covariates on time spent in MVPA/day among “old” participants aged 22–40.
**Table S3**. Association of AdjBW and select covariates by sex.

## Data Availability

The data that support the findings of this study are available from the corresponding author upon reasonable request.
