# Supplementary material for: Size at birth and accelerometer‐measured physical activity or sedentary behavior in healthy term‐born adults
Source: Am J Hum Biol. 2022 Jan 2;34(6):e23717. doi: 10.1002/ajhb.23717 (PMC9177509; doi:10.1002/ajhb.23717)
Supplement: Supplementary file 1 — Table S1 Association of AdjBW and select covariates on time spent in MVPA/day among “young” participants aged 18–21. Table S2. Association of AdjBW and select covariates on time spent in MVPA/day among “old” participants aged 22–40. Table S3. Association of AdjBW and select covariates by sex. [file AJHB-34-e23717-s001.docx]

Table S1. Association of AdjBW and select covariates on time spent in MVPA/day among “young” participants aged 18 – 21.

| Time spent in MVPA/day | B (95% CI) | p-value | Partial η^2^ |
| --- | --- | --- | --- |
| AdjBW | 7.02 (1.36, 12.68) | **.02** | .15 |
| Sex | -2.96 (-22.67, 16.74) | .76 | .00 |
| Age | -2.45 (-7.94, -3.04) | .37 | .02 |
| Body fat percentage | 1.04 (0.14, 1.94) | **.02** | .14 |
| VO_2Max_ (ml/kg/min) | 1.63 (0.79, 2.45) | **≤.001** | .31 |
| Wear Time | -0.05 (-0.11, 0.02) | .18 | .05 |

N = 42

Overall R^2^ = 0.49 (Adjusted R^2^ = 0.40)

Table S2. Association of AdjBW and select covariates on time spent in MVPA/day among “old” participants aged 22 – 40.

| Time spent in MVPA/day | B (95% CI) | p-value | Partial η^2^ |
| --- | --- | --- | --- |
| AdjBW | -10.77 (-19.81, 1.73) | **.02** | .19 |
| Sex | -2.80 (-25.24, 19.64) | .80 | .00 |
| Age | -0.65 (-2.12, 0.81) | .37 | .03 |
| Body fat percentage | 0.12 (-1.25, 1.48) | .86 | .00 |
| VO_2Max_ (ml/kg/min) | 1.72 (0.76, 2.68) | **.001** | .34 |
| Wear Time | 0.03 (-0.06, 0.12) | .49 | .02 |

N = 33

Overall R^2^ = 0.53 (Adjusted R^2^ = 0.42)

Table S3. Association of AdjBW and select covariates by sex.

|  |  | Females  (N = 60) | | | Males  (N = 15) | | |
| --- | --- | --- | --- | --- | --- | --- | --- |
|  |  | B  (95% CI) | p-value | Partial η^2^ | B  (95% CI) | p-value | Partial η^2^ |
| Time spent in MVPA | AdjBW | 5.43  (-1.79, 12.64) | .14 | .04 | 9.10  (-11.24, 29.44) | .33 | .12 |
|  | Age | -1.31  (-2.26, -0.36) | **.01** | .13 | 0.55  (-2.84, 3.93) | .72 | .02 |
|  | VO_2Max_ (ml/kg/min) | 1.59  (0.92, 2.26) | **≤.001** | .30 | 1.50  (-0.03, 3.03) | .05 | .39 |
|  | AdjBW * age | -0.69  (-1.67, 0.29) | .16 | .04 | -2.30  (-4.72, 0.13) | .06 | .37 |
| Time spent in SED | AdjBW | -13.46  (-36.68, 9.77) | .25 | .02 | 26.73  (-17.35, 70.81) | .20 | .17 |
|  | Wear Time | 0.52  (0.28, 0.76) | **≤.001** | .26 | 0.81  (0.06, 1.55) | **.04** | .40 |
|  | VO_2Max_ (ml/kg/min) | -3.47  (-6.24, -0.70) | **.02** | .10 | -1.53  (-6.52, 3.46) | .51 | .05 |

MVPA: Overall R^2^ = 0.39; Adjusted R^2^ = 0.32 (females);

Overall R^2^ = 0.58; Adjusted R^2^ = 0.27 (males)

SED: Overall R^2^ = 0.35; Adjusted R^2^ = 0.29 (females);
Overall R^2^ = 0.54; Adjusted R^2^ = 0.28 (males)
